# Sonothrombolysis in the ambulance for ST-elevation myocardial infarction: rationale and protocol

**DOI:** 10.1007/s12471-020-01516-9

**Published:** 2020-11-12

**Authors:** S. El Kadi, T. R. Porter, A. C. van Rossum, O. Kamp

**Affiliations:** 1 Department of Cardiology, Amsterdam Cardiovascular Sciences, Amsterdam UMC Location VUMC, Amsterdam, The Netherlands; 2 Division of Cardiovascular Medicine, University of Nebraska Medical Center, Omaha, NE USA

**Keywords:** Sonothrombolysis, ST-elevation myocardial infarction, Ambulance, Treatment

## Abstract

**Background:**

Treatment of ST-elevation myocardial infarction (STEMI) has improved over the years. Current challenges in the management of STEMI are achievement of early reperfusion and the prevention of microvascular injury. Sonothrombolysis has emerged as a potential treatment for acute myocardial infarction, both for epicardial recanalisation as well as improving microvascular perfusion. This study aims to determine safety and feasibility of sonothrombolysis application in STEMI patients in the ambulance.

**Methods:**

Ten patients with STEMI will be included and treated with sonothrombolysis in the ambulance during transfer to the PCI centre. Safety will be assessed by the occurrence of ventricular arrhythmias and shock during sonothrombolysis intervention. Feasibility will be assessed by the extent of protocol completion and myocardial visibility. Efficacy will be determined by angiographic patency rate, ST-elevation resolution, infarct size and left ventricular volumes, and function measured with cardiovascular magnetic resonance imaging, and contrast and strain echocardiography. A comparison will be made with matched controls using an existing STEMI database.

**Discussion:**

Sonothrombolysis is a novel technique for the treatment of cardiovascular thromboembolic disease. The first clinical trials on its use for STEMI have demonstrated promising results. This study will be the first to examine the feasibility of in-ambulance sonothrombolysis for STEMI.

**Trial registration:**

EU Clinical Trials Register (identifier: 2019-001883-31), registered 2020-02-25.

## Background

ST-segment elevation on the electrocardiogram (ECG) indicates the occlusion of an epicardial coronary artery, most commonly due to thrombus formation at the site of a ruptured atherosclerotic plaque. The optimal treatment strategy for ST-elevation myocardial infarction (STEMI) is immediate restoration of epicardial coronary blood flow. Currently, primary percutaneous coronary intervention (PCI) with concomitant dual antiplatelet therapy and periprocedural anticoagulation constitutes the treatment of choice in STEMI patients [1]. While successful reperfusion has led to a marked decrease in annual deaths over the past decades, long-term mortality and morbidity remain substantial [2, 3]. In addition to prolonged ischaemia and subsequent infarct size, microvascular injury due to ischaemic and reperfusion processes is a major determinant of adverse left ventricular remodelling and poor prognosis [4] and occurs in up to 50% of the patients [5]. By minimising infarct size and microvascular injury, left ventricular function may be preserved with improved long-term prognosis. Several studies have focused on early therapeutic interventions for reduction of infarct size and microvascular injury. Early drug therapy strategies include the use of low-dose fibrinolysis [6, 7], beta-blockers [8], adenosine [9], cyclosporine [10] and complement inhibition [11]. No or only small beneficial effects in a selected patient population have been demonstrated. Other interventions such as thrombus aspiration to reduce distal embolisation [12] and remote ischaemic conditioning [13] to reduce the possible detrimental effects of spontaneous reperfusion likewise did not improve outcome. A growing number of studies on adjunctive therapies for STEMI are performed in the pre-hospital setting. The time from STEMI diagnosis to PCI provides a window of opportunity for early therapy. A summary of recent studies on pre-hospital therapeutic strategies before PCI to reduce infarct size in STEMI patients and improve clinical outcomes is listed in Tab. 1. Recently, sonothrombolysis has been proposed as an adjunctive therapy for acute myocardial infarction [14]. In-vitro studies and in-vivo STEMI models have shown the potential of sonothrombolysis for restoration of epicardial and microvascular flow [15]. A recent randomised controlled trial on the effects of pre- and post-PCI sonothrombolysis in STEMI patients demonstrated a higher angiographic recanalisation rate prior to PCI and reduced infarct size in the intervention group [16]. Pre-PCI sonothrombolysis was performed at the emergency department in the hospital and resulted in thrombolysis in myocardial infarction (TIMI) flow grade ≥2 in almost half of the patients. By performing sonothrombolysis even before hospital arrival, its efficacy may be further improved. In this study, safety and technical feasibility of sonothrombolysis in STEMI patients in the pre-hospital ambulance setting will be assessed. To our knowledge, this is the first time that therapeutic echocardiography will be used in the ambulance.

**Table 1:** Summary of recent randomised controlled trials on pre-hospital therapeutic strategies before PCI in patients with acute myocardial infarction

Therapy	Study	No. of patients	Primary endpoints	Outcome
Abciximab	Pels et al. (2008) [34]	101	STR before PCI Initial TIMI 3 flow in IRA	No improvement in initial TIMI flow or STR
	Petronio et al. (2012) [35]	110	Infarct size (LGE-MRI)	No reduction in infarct size
	Ohlmann et al. (2012) [36]	256	Complete STR (>70%) after PCI	No improvement in STR
Tirofiban	Van ’t Hof (2008) [37]	984	Mean residual STD after PCI	Improvement in STR
	El Khoury et al. (2010) [38]	320	Initial TIMI 2/3 flow in IRA	No improvement in initial TIMI 2/3 flow
Clopidogrel	Zeymer et al. (2012) [39]	337	Initial TIMI 2/3 flow in IRA	No improvement in initial TIMI 2/3 flow
Ticagrelor	Montalescot et al. (2014) [40]	1862	Initial TIMI flow STR before PCI	No improvement in initial TIMI flow or STR
Bivalirudin	Steg et al. (2013) [41]^a^	2218	Combined endpoint of death or major bleeding (30 days)	Reduced composite endpoint (driven by less major bleeding), increased stent thrombosis
Tenecteplase	Armstrong (2006) [42]	304	Combined endpoint of death, re-infarction, refractory ischaemia, congestive heart failure, cardiogenic shock, and major ventricular arrhythmia	No difference in combined endpoint compared with PCI alone
	Thiele et al. (2011) [43]	162	Infarct size (LGE-MRI)	No reduction in infarct size
Metoprolol	Ibanez et al. (2013) [44]^a^	270	Infarct size (LGE-MRI)	Reduction in infarct size
	Roolvink et al. (2016) [8]	684	Infarct size (LGE-MRI)	No reduction in infarct size
Oxygen	Stub et al. (2015) [45]	441	Infarct size (enzymes)	Increase in infarct size
	Khoshnood et al. (2018) [46]	95	Myocardial salvage index (LGE-MRI)	No improvement of myocardial salvage
Ischaemic conditioning	Hausenloy et al. (2019) [47]^a^	5115	Combined endpoint of cardiac death and HF hospitalisation	No improvement of clinical outcomes
Hypothermia	Testori et al. (2019) [48]	101	Myocardial salvage index (LGE-MRI)	No improvement of myocardial salvage

*LGE-MRI* late gadolinium enhancement—magnetic resonance imaging, *TIMI* thrombolysis in myocardial infarction, *STR* ST-segment resolution, *STD* ST-segment deviation, *PCI* percutaneous coronary intervention, *IRA* infarct-related artery, *MACE* major adverse cardiac events, *HF* heart failure

^a^Some patients received treatment during transfer to the PCI centre, others received the treatment upon arrival in the hospital

## Methods

### Objectives

The primary objective is to study safety and feasibility of sonothrombolysis application in the ambulance. The secondary objective is to evaluate the efficacy of in-ambulance sonothrombolysis as assessed by ST-elevation resolution, initial angiographic patency, infarct characteristics and left ventricular dimensions, function, perfusion and remodelling.

### Endpoints

The main endpoints are safety and practical feasibility. Safety will be assessed by the occurrence of ventricular arrhythmias defined as sustained ventricular tachycardia and/or ventricular fibrillation and the occurrence of shock defined as a systolic blood pressure <100 mm Hg in combination with tachycardia (heart rate >100/min), after initiation of sonothrombolysis and before PCI.

These endpoints will provide relevant information regarding the safety of sonothrombolysis in the ambulance. Two forms of shock are relevant for the present study: anaphylactic shock (very rare in case of commercially available microbubbles, 1:10,000–100,000) and cardiogenic shock. The incidence of cardiogenic shock in STEMI patients is around 7% [17]. The incidence of ventricular arrhythmias in STEMI patients before percutaneous coronary intervention is around 3% [18]. It is important to note that some ventricular arrhythmias, e.g. runs of ventricular premature complexes or accelerated idiopathic ventricular arrhythmias, are not uncommon after flow restoration in STEMI and are even related to improvement of myocardial perfusion [19].

Practical feasibility will be assessed by the extent of sonothrombolysis completion during ambulance transfer. The ultrasound images will be saved for offline review; sonothrombolysis completion will be measured by counting the number of high mechanical index impulses applied in each view and the quality of the acquired apical views will be scored.

Secondary endpoints are ST-elevation resolution, initial TIMI flow, infarct characteristics and left ventricular dimensions and function at baseline (3–5 days) and follow-up (6–8 weeks) with cardiovascular magnetic resonance imaging (CMR). Myocardial perfusion and left ventricular remodelling parameters will be assessed on follow-up contrast-enhanced echocardiography (3–4 months). Six-month event-free survival will be calculated from treatment initiation to 6 months afterwards, where events include death, congestive heart failure, life-threatening arrhythmias, recurrence of acute coronary syndrome and need for prophylactic defibrillator.

### Patient enrolment

This study will be conducted in accordance with the standards for Good Clinical Practice and the Declaration of Helsinki. Ethics approval was obtained on 3 February 2020 from the Medical Ethics Review Committee VUmc (reference: 2019.556—NL69980.029.19). Given the logistical challenge of the study and the primary aim to assess feasibility, a total of ten patients will be included. Due to the urgent setting and need for rapid initiation of the study protocol, only brief oral consent will be obtained. The written informed consent procedure will be deferred until after coronary angiography. Inclusion and exclusion criteria are listed in Tab. 2. All patients will receive drug therapy consisting of dual antiplatelet therapy (DAPT) and intravenous heparin in the ambulance. DAPT will be continued for 1 year according to current guidelines.

**Table 2:** Eligibility criteria

Inclusion criteria	– Acute (within 12 h) or worsening chest pain or shortness of breath associated with >1.0 mV ST elevation on the ECG in at least 2 contiguous leads – Age ≥30 years – Adequate apical and/or parasternal images by echocardiography
Exclusion criteria	– Previous coronary bypass surgery – Cardiogenic shock – Known or suspected hypersensitivity to ultrasound contrast agent used for the study – Known bleeding diathesis or contraindication to glycoprotein IIB/IIIA inhibitors, anticoagulants or aspirin – Known large right to left intracardiac shunts

### Study procedures

**Fig. 1:**
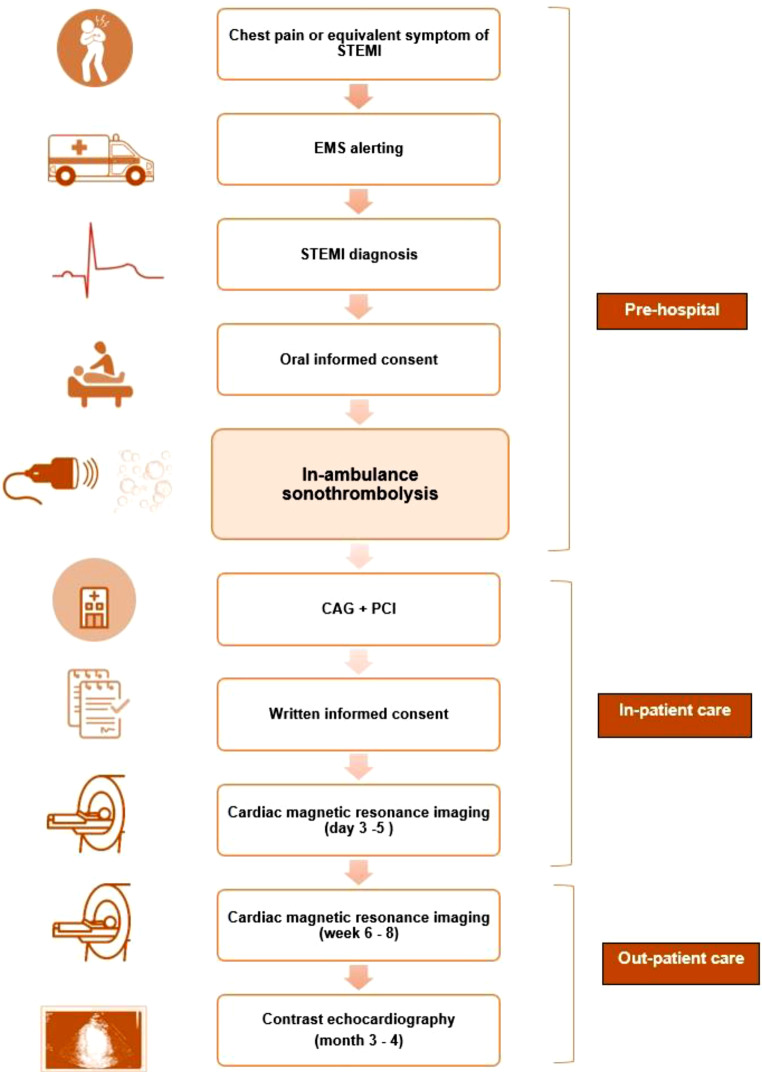
Study flow chart. (*STEMI* ST-elevation myocardial infarction, *EMS* emergency medical services, *CAG* coronary angiography, *PCI* percutaneous coronary intervention)

**Fig. 2:**
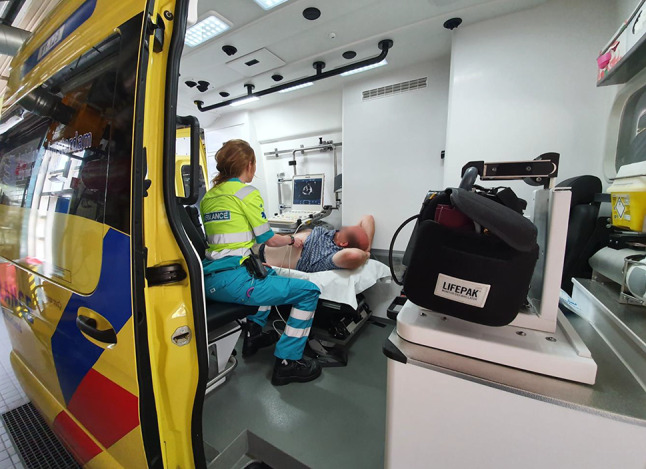
Portable ultrasound system mounted inside the ambulance enables diagnostic and therapeutic echocardiography (sonothrombolysis) during patient transfer to the PCI centre

The study will be conducted by the Amsterdam University Medical Center—location VUmc in collaboration with the emergency medical services (EMS) in Zaandam, the Netherlands. In the ambulance, ECGs of patients with signs of STEMI will be faxed to the PCI centre via the Lifenet system [20]. When STEMI diagnosis is confirmed and patient is accepted for coronary angiography with optional PCI, eligible patients will be asked to give oral informed consent and will be included in the trial (Fig. 1, Study flow chart). Sonothrombolysis will be performed by a trained medical doctor (SK) in the ambulance and applied for 20 min or until hospital arrival. The treatment consists of commercially available microbubble infusion (1.5 ml Luminity, diluted in 50 ml NaCl; Lantheus MI, Newbury, United Kingdom) and simultaneous transthoracic echocardiography with a Philips CX-50 ultrasound machine built into the ambulance (Fig. 2). Low mechanical index imaging (MI <0.3) will be used to visualise the myocardium within each view and to monitor for replenishment within each of the segments after application of high MI. Multiple (>10) intermittent high MI impulses (20 frames ~1.0–1.2 MI; transmit frequency 1.6 MHz; pulse duration <5 µs) will be administered in either three apical windows or parasternal short-axis windows, depending on image quality. A total of 30–60 impulses will be given, with 4–8 sec between consecutive high MI impulses to allow for replenishment of contrast within the perfusion beds. The total duration of sonothrombolysis will be measured as well as total number of applied flashes in each view. Off-site quality analysis of the images will be performed in which the in-plane visibility of the myocardium will be recorded and scored by an experienced blinded core lab.

At least three ECGs will be performed: before and after sonothrombolysis and after primary PCI. Absolute and relative cumulative ST-elevation resolution will be measured. Creatine kinase (CK), creatine kinase MB (CK-MB) and troponin levels will be obtained through standard care venous blood sampling in the hospital. Maximum biomarker levels will be reported.

All patients will undergo invasive coronary angiography. Primary PCI will be performed according to the guidelines and at the discretion of the interventional cardiologist. TIMI flow grade before and after PCI, myocardial blush grade and thrombus burden will be reported.

CMR imaging will be performed at baseline (3–5 days after presentation) and 6 to 8 weeks later. A clinical 1.5 or 3.0 T scanner with phased-array cardiac receiver coils will be used. Cine images are obtained using a steady state free precession sequence in three long-axis views and multiple short-axis views to examine left ventricular mass, function and volumes and to quantify left ventricular ejection fraction. T2-weighted images will be taken in short-axis planes identical to the cine images. T1 mapping in three parallel short-axis views positioned at the basal, mid and apical levels will be done using a Shortened Modified Look-Locker Inversion recovery sequence. First-pass perfusion will be performed in the same three short-axis views as T1 mapping using saturation recovery imaging. Early and late gadolinium enhanced (LGE) images are acquired 2 and 10 min, respectively, post-injection using a segmented gradient echo sequence. LGE images will be used to assess infarct size, the presence and extent of microvascular injury and to measure the area at risk. Short-axis T2-weighted images will be used for the detection of intramyocardial haemorrhage and infarct-related oedema. All CMR analyses will be performed in a core laboratory by blinded observers.

Contrast-enhanced echocardiography will be performed 3–4 months after initial presentation. Left ventricular function and volumes will be measured. Left ventricular remodelling will be defined as a 20% increase in end-diastolic volume at the 3 month follow-up biplane contrast-enhanced echocardiogram compared with the pre-discharge echocardiogram.

Follow-up will take place at 6 months by telephone contact and electronic patient records will be consulted.

### Statistical analysis

Statistical analyses for the primary endpoints will be purely descriptive. Baseline characteristics will be presented, continuous variables will be given as *n* (%) and mean with interquartile range (IQR). Secondary outcomes will be compared between patients included in this study and historical control patients. All these analyses will be considered exploratory. Categorical variables will be compared between groups using a chi-square test or Fisher’s exact test. Continuous variables will be compared using an independent sample t‑test (if normally distributed) or the Mann-Whitney test (otherwise). The study and the trial conduct will be audited by an independent monitor from the Amsterdam UMC.

### Challenges

Although multiple studies on new therapeutic strategies for STEMI have been performed in the pre-hospital setting (Tab. 1), incorporation of sonothrombolysis in the ambulance is more challenging.

It requires preparation of all materials and processes, briefing of EMS nurses and staff of the emergency control room as well as the presence of a trained physician to perform sonothrombolysis. The aim is to complete the entire process—from oral informed consent and microbubble preparation to ultrasound imaging and sonothrombolysis—during patient transfer. Obtaining oral consent, preparing the microbubble solution and obtaining appropriate ultrasound views is estimated to take 4 min. The ultrasound regimen consists of 30–60 high MI impulses and can be completed within 8 min, considering a 4–8 sec microbubble replenishment time in between the high MI impulses. To prevent any delay in patient transfer, the study treatment needs to be completed within 10–15 min, which approximates the mean duration of the ambulance ride to the PCI centre. A custom-built holder has been developed to keep the ultrasound machine in place (Fig. 2). Furthermore, since Luminity microbubbles will be used, a mobile cooling device is necessary to comply to the recommended storage conditions. Since only one vehicle is equipped with the appropriate sonothrombolysis equipment, recruitment time might be long. To overcome this difficulty, the ambulance control room will be notified about the study and requested to assign patients with possible STEMI (e.g. patients with chest pain) to the equipped vehicle.

## Discussion

Thrombolysis and PCI have significantly improved the prognosis of STEMI patients. However, two major clinical problems remain. Firstly, early reperfusion is restricted by patient factors, delays in transfer to PCI capable centres and delays in PCI initiation [1]. The longer the ischaemic time, the greater the size and transmurality of the infarct zone [21, 22]. Actions have been taken to reduce ischaemic time by facilitating early STEMI diagnosis and prehospital activation of the cardiac catheterisation lab. However, total ischaemic time in STEMI patients remains substantial [23]. Secondly, although successful epicardial revascularisation may be achieved, up to 50% of the patients exhibit microvascular injury, resulting in higher infarct sizes, adverse left ventricle remodelling and worse prognosis [4]. Novel therapeutic interventions for STEMI should preferably be applied within minutes of STEMI diagnosis and should also treat or prevent microvascular injury.

**Fig. 3:**
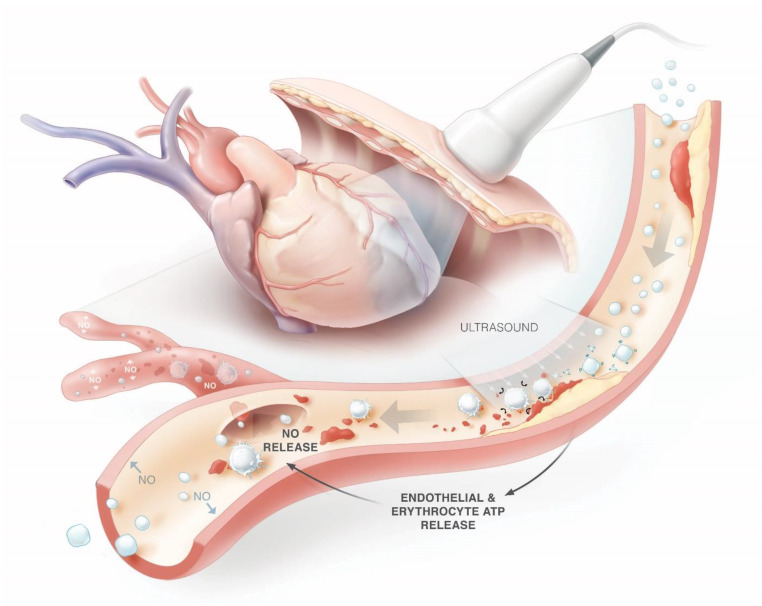
Restoration of epicardial and microvascular blood flow in ST-elevation myocardial infarction using sonothrombolysis. (NO nitric oxide, ATP adenosine triphosphate)

In recent years, sonothrombolysis has emerged as a potential adjunctive therapy in cardiovascular ischaemic disease. The technique is based on the lytic and flow-inducing properties of the ultrasound-induced cavitation of contrast microspheres. Once high MI ultrasound impulses are administered during simultaneous infusion of ultrasound contrast agents, the contrast microspheres compress and expand and eventually collapse, inducing local microstreaming capable of thrombus dissolution (Fig. 3; [24, 25]). Various animal models have been used to examine therapeutic contrast echocardiography with and without fibrinolytic drugs [26–28]. Xie et al. found unexpectedly that high MI impulses using a diagnostic transducer in combination with microbubbles in pigs with acute left anterior descending occlusions not only resulted in higher epicardial recanalisation rates but also resulted in ST-segment resolution and corresponding recovery of wall thickening in six pigs (40%) that did *not* have epicardial recanalisation [29]. Improvement of microvascular flow could be related to sonolysis of microvascular thrombi or the increased release of the vasodilator and anti-inflammatory nitric oxide from the microvascular endothelium (Fig. 3). The latter explanation is supported by a study in mice where augmentation of limb perfusion was observed upon ultrasound and microbubbles application, whereas inhibition of endothelial nitric oxide synthase attenuated the flow augmentation by 70% [30]. The first pilot study on clinical application of ultrasound and microbubbles in STEMI patients showed safety and feasibility of pre-PCI sonothrombolysis [31]. As preclinical studies suggested efficacy of thrombus dissolution could be improved by increasing high MI pulse duration [32], a small trial was performed using 20 µs high MI impulses [33]. While feasible for restoring microvascular perfusion, transient focal vasoconstriction was seen in three of six patients. In the subsequent MRUSMI trial, 100 patients were randomised to either diagnostic ultrasound-guided treatment with intermittent high MI impulses (5 µs) during microbubble infusion or to a control group [16]. Treatment was given pre- and post-PCI and lasted approximately 15 min per treatment. Angiographic recanalisation prior to PCI was achieved in 48% in the treatment group versus 20% in the control group. Positive results were observed regarding infarct size, left ventricular ejection fraction and need for defibrillator implantation, while vasoconstriction was not observed. Currently, several trials are ongoing that aim to investigate the effects of post-PCI sonothrombolysis on microvascular injury and infarct size (MRUSMI, EU Clinical Trials Register 2018-001277-24).

Since contrast ultrasound is portable, readily available and non-invasive, this technique can be provided at the point-of-care to the STEMI patient. To prevent system delay, sonothrombolysis in the ambulance may offer a convenient solution to achieve early myocardial reperfusion and concomitantly treat microvascular injury in the setting of a STEMI. This study will be the first study in the ambulance setting to examine feasibility of pre-hospital therapeutic contrast ultrasound in STEMI patients.

## Trial status

Recruitment is expected to start in September 2020, completion of recruitment is expected in December 2020. The date and version of the study protocol approved by the ethical review board are: version 3, January 2020.
